# Attitude of Medical Students Toward Rising Violence Against Doctors

**DOI:** 10.7759/cureus.75078

**Published:** 2024-12-04

**Authors:** Arya Jith, Anju Lakshmi, Sharon Methala, Kathleen A Mathew

**Affiliations:** 1 Psychiatry, Sree Uthradom Thirunal (SUT) Academy of Medical Sciences, Thiruvananthapuram, IND; 2 Psychiatry, Amrita Institute of Medical Sciences, Kochi, IND; 3 Gastroenterology, Amrita Institute of Medical Sciences, Kochi, IND

**Keywords:** attitude, awareness, medical students, violence, violence against doctors

## Abstract

Introduction: In the past three years, there has been an increase in incidents of violence against healthcare workers in Kerala. The aim of the study is to explore the attitude of medical students toward violence against doctors.

Methodology: This was a cross-sectional study undertaken at three of the four medical colleges in Ernakulam district in India as a part of our convenience sampling, which included around 1,500 students. Students from first-year Bachelor of Medicine, Bachelor of Surgery (MBBS) to students doing internships were included in the study. The authors prepared a semi-structured questionnaire assessing the attitude of medical students toward rising violence against doctors, which was circulated among the students.

Results: A total of 347 medical students participated in the study. Of these, 338 (97.4%) expressed concern about such incidents. 248 (71.5%) reported that emergency medicine doctors were more prone to such violence than any other specialties, and 221 (63.7%) of the participants preferred to go abroad for higher studies and continue their careers there. Two hundred ten (60.5%) of the students were not aware of any legal provision to safeguard doctors from workplace violence. Primary contributing factors to violence were “unrealistic expectations of the family from the doctors,” “poor communication skills of the doctor,” “inadequate time spent by the doctor in explaining the prognosis,” and “not taking proper action against the violators.”

Conclusion: In order to prevent such incidents, changes should be made at various levels. Adequate training should be done to improve soft skills, including the communication skills of doctors. Adequately trained security staff should be provided at the hospital, especially the casualty. The government should make a stringent central law that protects hospitals and healthcare staff.

## Introduction

The World Health Organization (WHO) defines workplace violence as “incidents where staff is abused, threatened, or assaulted in the circumstances linked to their workplace, including commuting to and from the workplace, involving an explicit or implicit challenge to their safety, well-being or health.” Compared to all other professions, healthcare workers have a four times higher risk of violence in the workplace. It can be in the form of physical violence, psychological violence, or a combination of both [1].

The earliest studies of violence against doctors are from the US, which dates back to the late 1980s. In these studies, 57% of the emergency care staff reported that they had been threatened with a weapon, whereas in the UK, 52% of doctors reported some kind of violence [2]. In Asia, the highest rates of violence against medical professionals have been reported from China. Prevalence rates of violence against health professionals in countries like India, Israel, Pakistan, and Bangladesh have been higher when compared to the rates in Western countries [3].

In India, nearly 75% of doctors report having dealt with one or the other form of violence during their practice. Almost 50% of these incidents were reported in intensive care units (ICUs), and in 70% of the cases, the relatives of patients were actively involved in such incidents [4]. As per the Indian Medical Association (IMA), at least five cases of attacks on doctors are reported every month in Kerala, and more than 200 such attacks, including intimidation and threats, have been reported in the past three years [5]. Recently, a young doctor was stabbed to death by an intoxicated patient who was brought to the emergency services by police officers for medical examination [6].

Violence against doctors is a growing concern among the medical fraternity. Medical students are the clinicians of tomorrow. Hence, their perception regarding rising incidents of violence against medical professionals needs to be assessed. As per our literature review, although few studies were done among practicing doctors, there needed to be studies assessing the attitude of budding doctors toward such incidents in India.

In this study, we aim to explore the perspective of medical students with regard to the increasing occurrence of violence against doctors and their concerns regarding the same. Hence, the objectives of the study include discussing the awareness of medical students toward rising violence against doctors, factors contributing to violence against doctors, and the attitude of medical students toward increasing violence against doctors.

## Materials and methods

This was a cross-sectional study done among undergraduate medical students and interns in Ernakulam district in India during a period of three months (August 2021-October 2021). This study was approved by the Institutional Review Board of Amrita Institute of Medical Sciences and Research Centre, Kochi, with approval number ECASM-AIMS-2021-322, dated 27-07-2021. The study was carried out in accordance with the principles as enunciated in the Declaration of Helsinki.

There are 30 medical colleges across Kerala, and Ernakulam has four medical colleges. We have selected three medical colleges at Ernakulam as a part of our convenience sampling, and these three medical colleges have around 1,500 students. All medical students from their first year till their internship studying in these medical colleges were included in the study. Participants who did not give consent were excluded from the study. The sample size was not calculated since we have included all medical students in these three medical colleges.

As per our literature review, there is a scarcity of studies on this topic, and we could not find any validated questionnaire. A semi-structured questionnaire was prepared based on a questionnaire used in a previous study [7]. We also conducted five focused group discussions (FDGs) on the students from the same institution. A total of 50 medical students participated. The points discussed were noted, and a few themes were identified, such as awareness, factors for violence, and the attitude of the students toward such incidents. Questions included in the questionnaire were based on these themes. The questionnaire had 21 questions and was divided into three sections. The first part included questions assessing sociodemographics and awareness regarding the incidents, the second part included variables assessing various factors that lead to such incidents, and the third part included the attitude of medical students toward rising violence (questionnaire in the Appendix).

The questionnaire, prepared using Google Forms (Google LLC, Mountain View, California, United States), was circulated using platforms like WhatsApp and Facebook. Fifteen students from all three medical colleges were contacted, and the form was shared with their college mates. Informed online consent was obtained from all participants after providing details of the conduct of the study, assurance of participants' confidentiality, and voluntary participation.

The data analysis was carried out using IBM SPSS Statistics for Windows, version 20.0 (released 2011, IBM Corp., Armonk, NY). Continuous variables were expressed using mean ± SD, while categorical variables were represented as frequency and percentages.

## Results

Among 1,500 students, only 400 students (26.6%) attempted the questionnaire, and only 347 (23.1%) completed the questionnaire, among which the majority were females (258; 74.4%) and hailed from Kerala (306; 88.2%). The majority of the students who participated were doing their second year of medical training (108; 31.1%) and 243 (70.0%) did not have a medical background. The main reason for joining MBBS was out of self-interest (208; 59.9%) (Table 1).

**Table 1:** Frequency of various sociodemographic variables

Variables		Frequency	Percentage
Gender	Male	89	25.6
	Female	258	74.4
State hailing from	Kerala	306	88.2
	Other south Indian states	22	6.3
	North India	19	5.5
Currently pursuing	First year	63	18.2
	Second year	108	31.1
	Third year	44	12.7
	Final year	59	17.7
	House surgeon	73	21
Medical background in the family	Yes	104	30
	No	243	70
Reason for choosing MBBS	Parents' suggestion	47	13.5
	Societal status and high salary	19	5.5
	Self interest	208	59.9
	Serving humanity	73	21

Furthermore, 344 (99.1%) reported being aware of violence against doctors, and 338 (97.4%) expressed concern about such incidents. The majority of them reported social media as the source of information regarding such incidents (303; 87.3%). Three hundred (86.5%) reported that doctors are at a higher risk of workplace violence than any other profession, and 284 (81.8%) believed that self-defense training should be included in the MBBS curriculum. A total of 248 (71.5%) reported that emergency medicine doctors were more prone to such violence than any other specialties and 195 (56.2%) believed that it would be safer to opt for a non-clinical or para-clinical specialty for post-graduation than a clinical specialty (Table 2).

**Table 2:** Awareness of medical students regarding violence against doctors Surgery includes general surgery, orthopedics, gastro surgery, neurosurgery, ENT, and ophthalmology.

Variables		Frequency	Percentage
Awareness regarding violence against doctors	Yes	344	99.1
	No	3	0.9
Are you concerned about this violence?	Yes	338	97.4
	No	9	2.8
Source of information regarding these incidents	Social media	303	87.3
	Electronic media	35	10.1
	Print media	9	2.6
Do you feel doctors are at high risk?	Yes	300	86.5
	No	47	13.5
Do you feel self-defense training is necessary for doctors?	Yes	284	81.8
	No	63	18.2
Which specialty is prone to violence?	General medicine	19	5.5
	Emergency	248	71.5
	Surgery	52	15
	Anesthesia	2	0.6
	Pediatrics	5	1.4
	Gynecology	11	3.2
	Psychiatry	5	1.4
	All clinical fields equally	5	1.4
Do you feel it’s safer to opt for non/para-clinical subjects?	Yes	195	56.2
	No	152	43.8

Among patient-related factors for violence, 232 (66.9%) considered “unrealistic expectation of family from doctors’’ as the main contributing factor. “Inadequate time spent by the doctors in explaining the prognosis” was considered the most common clinician-related factor contributing to workplace violence. Among policymaker-related factors, 254 (73.2%) believed that “not taking proper action against violators when an incident happens” was the major factor leading to rising incidents of violence against doctors. Among hospital-related factors, 150 (43.2%) believed that “giving unrealistic expectations to patients and relatives“ was the main contributing factor to violence against doctors (Table 3).

**Table 3:** Frequency of various factors in detail that contribute to violence against doctors.

Variables	Variables	Frequency	Percentage
Patient attendant-related factors	Delay in treatment-seeking	44	12.7
	Poor knowledge regarding illness	49	14.1
	Negative attitude towards allopathic medicine	22	6.3
	Unrealistic expectations of family from doctors	232	66.9
Doctor-related factors	Poor communication skills of the doctor	152	43.8
	Doctors' incompetence in medical care	20	5.8
	Doctors' insensitive behaviour toward the family	17	4.9
	Inadequate time spent by the doctor in explaining the prognosis	158	45.5
Policymaker-related factors	Not taking proper action against violators when an incident is reported	254	73.2
	Not providing hospitals with adequate infrastructure	43	12.4
	Not making a protocol for the media to report such incidents	36	10.4
	Political hooliganism	14	4
Hospital-related factors	Giving unrealistic expectation to patients and relatives	150	43.2
	Corporatization of medical care with profit motive	116	33.4
	Not providing adequate security staff	81	23.3

Meanwhile, 221 (63.7%) of the participants preferred to go abroad for higher studies and continue their careers there. Two hundred fourteen (61.7%) suggest that the younger generation will take up this profession, and 249 (71.8%) will not opt for an alternative career if given a choice. Some (210; 60.5%) of the students were not aware of any legal provision to safeguard doctors from workplace violence. In addition, 287 (82.7%) believed that incidents of violence against doctors escalated after the COVID-19 pandemic, and 308 (88.8%) believed that such incidents would lead to more and more doctors adopting defensive medical practices (Table 4).

**Table 4:** Variables assessing the attitude of medical students toward rising violence against doctors.

Variables		Frequency	Percentage
Do you prefer working abroad for higher studies and jobs?	Yes	221	63.7
	No	126	36.3
Will you suggest the younger generation take up this profession?	Yes	214	61.7
	No	133	38.3
Will you opt for an alternative career if given a choice?	Yes	98	28.2
	No	249	71.8
Awareness regarding any laws related to the protection of doctors against violence?	Yes	137	39.5
	No	210	60.5
Do you think violence against doctors has escalated since the COVID-19 outbreak?	Yes	287	82.7
	No	60	17.3
Do you think violence will lead doctors to practice defensive medicine?	Yes	308	88.8
	No	39	11.2

## Discussion

Being a doctor was considered once a noble profession, and for a medical student, one would always dream about the respect attached to it. However, now, violence against doctors has become a global pandemic. In our survey, although we had circulated the questionnaire to all medical colleges, there was poor participation, and we had only 400 participants, of which only 347 (23.1%) completed the questionnaire. The majority of the students who participated were aware and concerned regarding the incidents of violence against doctors at the workplace. In our study, most participants were females since, in Kerala, girls always outnumbered boys in taking MBBS admission over the last five years [8]. The majority, 208 (59.9%) of the participants, took this course out of self-interest, and 73 (21%) opted for this profession to serve humanity.

A survey by the IMA reported that 82.7% of doctors felt stressed out by their profession, and 46.3% feared workplace violence. Many of those who are attacked or threatened reported to have experienced anger, fear, anxiety, self-blame, and loss of confidence. There were many incidents reported in India where doctors committed suicide after such allegations of medical negligence [9]. Majority (71.5%) believed that emergency medicine physicians are more prone to get attacked by bystanders. Surgery (15%) and general medicine (5%) were also considered high-risk specialty. These findings highlight the fact that students will start their medical careers with a guarded approach toward their patients. An internal analysis of the National Eligibility cum Entrance Test for Post Graduates (NEET PG) 2022 first counseling results for the first 500 all-India ranks showed that students opt more for nonsurgical subjects. The majority of the students opted for specialties like general medicine, radio-diagnosis, and dermatology while only very less students opted for obstetrics and general surgery. These findings are similar to a study done by Babu et al., which noted that the majority of students preferred to take radio-diagnosis and pediatrics as their specialty [10]. We assume that this could be because these specialties assure a high salary and are considered safer compared to others. General medicine and surgery were less preferred because of more hectic training and the need for superspecialty degrees for better pay and more risk of medical litigations. Contrary to this finding, general medicine was still the preferred specialty in NEET PG counseling; it might be because there is always a demand for general practitioners and could pave the way to explore many other areas of medicine. These assumptions require further empirical testing. In our survey, the majority (56.2%) felt that taking non/para-clinical subjects would have less risk of being attacked or threatened.

In our survey, to assess the reasons for violence, we have divided the questions into patient-related, hospital-related, doctor-related, policymaker-related, and administrator-related factors.

Among patient-related factors, "having unrealistic expectations of family from doctors" was considered one of the main reasons. Most of the time, when a patient gets better, the family believes that it’s God’s miracle, and when a patient dies, they believe that it is due to the doctor’s fault. Media might have a role in this since most of the time, when they report such deaths of patients, they allege it as medical negligence [11]. Recently, certain films have portrayed doctors as being involved in the "organ transplant mafia" and as being unconcerned about the well-being of patients. Such films and media reporting of deaths in the hospital as the result of conspiracies could give a wrong notion about doctors and hospitals [11-12].

Among doctor-related factors, “inadequate time spent by the doctors in explaining the prognosis of the illness to the family” was considered the main reason for violence. Most doctors are stressed due to heavy workloads, long working hours, and lack of adequate infrastructure, and hence, they spend less time explaining the prognosis of the patient to family members [13]. During medical training also, doctors are not taught how to break the bad news to the family members or how important is to spend time with the family in conveying the prognosis to the patient or family [14].

Among policymaker-related factors, “not taking proper actions against the violators when an incident is reported” was the main factor. Although there have been increasing incidents of violence against hospital staff, the government has not initiated any central law (no common CRPC/IPC section) against violators. Even though 19 state laws exist protecting hospital and their staff, most of the time, officials do not charge the case or compromise such incidents [15]. Recently, after the murder of a house surgeon on duty, the Kerala government has approved an ordinance aimed at protecting healthcare staff, which warrants up to seven years of jail imprisonment for attacking healthcare staff on duty and up to five lakhs rupees as a fine. The ordinance also prescribes that all cases registered under the law should be probed by an officer, not below the rank of inspector, and the probe should be completed within 60 days from the registration of the First Information Report (FIR) [16].

Among hospital-related factors, “giving unrealistic expectations to the family” was one of the reasons for violence. Most of the time, there is a lack of communication from the doctors, and the hospitals always portray their success stories in their advertisements, which gives a false sense of hope. With the advent of modern medicine, the cost of healthcare has increased globally. Still, due to low literacy rates in India, there is an unrealistic expectation that paying more money should save one's life, i.e., better outcomes are expected even for risky procedures [3].

Furthermore, 249 (71.8%) of the participants do not want to change their careers if given an alternate choice, and 214 (61.7%) will suggest the same profession to the younger generation. Of the participants, 221 (63.7%) would like to go abroad for higher education and a job. This could be because of better pay and less workload in Western countries [17]. The majority, 210 (60.5%), of the participants reported that they were not aware of any laws regarding hospital violence. Many, 308 (88.7%), reported that an increase in such incidents might lead to the practice of defensive medicine. Defensive medicine, in simple words, is departing from normal medical practice as a safeguard from litigation. Practicing defensive medicine is not good for patients or physicians. This is because doctors might advise doing certain diagnostic procedures for the confirmation of the diagnosis, which may add additional financial burden for the patients, and at the same time, they may also avoid doing certain risky procedures to avoid medical litigations [18].

Our study has various limitations. The number of students who participated in our study was very low compared to the total number of medical students in three medical colleges at Ernakulum; therefore, we cannot generalize our findings. The questionnaire that was used in our study was not a validated questionnaire.

## Conclusions

Due to rising rates of violence, there is an urgent need to make healthcare facilities safe for doctors, as only then they can work with complete dedication. This needs to be done at various levels by the government, media, and medical professionals alike. Every doctor should follow the cardinal principle “do not overreach,” i.e., do not treat beyond the scope of one's training and facilities to prevent violence and litigations against themselves. Doctors cannot be held accountable for every death that occurs in the hospital on account of negligence. The government should make a stringent central law that protects hospitals and healthcare staff. 

## Appendices

**Table 5:** Questionnaire of the study

Questions	Options
1. Gender	a. Male b. Female
2. State hailing from	a. Kerala b. Other south Indian states c.North India
3. Currently pursuing	a. First year b. Second year c. Third-year d. Final Year e. House surgeon
4. Medical background in the family	a. Yes b. No
5. Reason for choosing MBBS	a. Parent Suggestion b. Societal Status and high salary c. Self interest d. Serving Humanity
6. Awareness regarding violence against doctors	a. Yes b. No
7. Are you concerned about this violence?	a. Yes b. No
8. Source of information regarding these incidents	a. Social Media b. Electronic media c. Print media
9. Do you feel doctors are at high risk?	a. Yes b. No
10. Do you feel that self-defense training is necessary for doctors	a. Yes b. No
11. Which specialty is prone to violence?	a. General Medicine b. Emergency c. Surgery d. Anaesthesia e. Paediatrics f. Gynaecology g. Psychiatry
12. Is it safer to opt for non/para-clinical subjects?	a. Yes b. No
13. Patient-related factors	a. Delay in treatment seeking b. Poor knowledge regarding illness c. Negative attitude towards allopathic medicine d. Unrealistic expectation of family from doctors
14. Doctor-related factors	a. Poor communication skills of the doctor b. Doctors incompetence in medical care c. Doctors insensitive behaviour towards family d. Inadequate time spent by doctor in explaining the prognosis
15. Policy-related factors	a. Not taking proper action against violators when an incident is reported. b. Not providing hospitals with adequate infrastructure. c. Not making a protocol for media to reports such incidents.
16. Hospital-related factors	a. Giving unrealistic expectation to patient and relatives b. Corporatization of medical care with profit motive c. Not providing adequate security staff
17. Do you prefer working abroad for higher studies and job?	a. Yes b. No
18. Will you suggest the younger generation to take up this profession?	a. Yes b. No
19. Awareness regarding any laws related to the protection of doctors against violence?	a. Yes b. No
20. Do you think violence against doctors escalated since the COVID-19 outbreak?	a. Yes b. No
21. Do you you think such incidents will lead doctors to practice defensive medicine?	a. Yes b. No
